# SIRT1 regulates dermal fibroblast senescence via impaired deacetylase function and mitochondrial dysfunction during skin aging induced by chronic oral cadmium exposure

**DOI:** 10.3389/fpubh.2026.1779372

**Published:** 2026-02-24

**Authors:** Dehui Zhou, Gengsheng Yu, Xiaohui Fu, Qunchao Su, Chu Wang, Yonggang Ma, Hui Zou, Di Ran, Zongping Liu

**Affiliations:** 1 College of Veterinary Medicine, Yangzhou University, Yangzhou, Jiangsu, China; 2 Jiangsu Co-innovation Center for Prevention and Control of Important Animal Infectious Diseases and Zoonoses, Yangzhou, Jiangsu, China; 3 Joint International Research Laboratory of Agriculture and Agri-Product Safety of the Ministry of Education of China, Yangzhou University, Yangzhou, Jiangsu, China; 4 College of Veterinary Medicine, Southwest University, Chongqing, China

**Keywords:** acetylation, cadmium, dermal fibroblast, mitochondrial dysfunction, SIRT1, skin aging

## Abstract

**Introduction:**

Skin aging is a complex, multifactorial biological process that can be significantly accelerated by environmental toxicants such as cadmium (Cd), a highly toxic and ubiquitous heavy metal. Although the broad cytotoxic impacts of Cd have been extensively reported, a comprehensive understanding of the precise molecular pathways underlying Cd-induced skin senescence is still lacking. In this study, we investigated the protective role of Sirtuin 1 (SIRT1), a highly conserved nicotinamide adenine dinucleotide (NAD^+^)-dependent deacetylase that functions as a master regulator of mitochondrial homeostasis and cellular defense mechanisms.

**Methods:**

To delineate the influence of SIRT1 on dermal aging, we established an *in vitro* model using primary rat dermal fibroblasts and C3H/10 T1/2 cells, where SIRT1 levels were modulated via lentiviral-mediated overexpression. Concurrently, an *in vivo* model was developed using Sprague–Dawley rats subjected to chronic Cd exposure via drinking water (50 mg/L) for 6 months, complemented by skin-targeted SIRT1 upregulation through the local injection of AAV-r-SIRT1.

**Results:**

Our results demonstrate that Cd exposure elevates reactive oxygen species (ROS), disrupts mitochondrial integrity, and activates DNA damage responses, collectively driving cellular senescence. SIRT1 was shown to exert protective effects through the deacetylation of key substrates such as P53 and SOD2, thereby restoring redox balance and promoting DNA repair. The elevation of SIRT1 expression markedly mitigated mitochondrial impairments, senescent phenotypes, and apoptotic features triggered by Cd exposure.

**Conclusion:**

Our findings position SIRT1 as a crucial regulator of Cd-induced skin aging and suggest that targeting this deacetylase may provide a viable strategy to counteract skin degeneration caused by environmental insults.

## 1 Introduction

As a ubiquitous environmental contaminant, Cadmium (Cd) is predominantly discharged into the ecosystem via diverse industrial activities, notably mineral extraction, the manufacturing of batteries, and the utilization of phosphate-based fertilizers (1, 2). Owing to its prolonged biological persistence and tendency for bioaccumulation, Cd presents substantial health hazards and has been officially categorized as a Group 1 human carcinogen (3). Chronic Cd exposure has been associated with nephrotoxicity, carcinogenesis, and osteotoxicity, and recent evidence implicates it in age-associated cellular degeneration through redox disruption and mitochondrial damage (4, 5).

Because it functions as the major contact surface for external stressors, the skin is especially prone to damage mediated by Cd exposure (6). Cd can infiltrate dermal layers through direct contact or systemic circulation, leading to oxidative stress, inflammation, and impaired repair mechanisms (7). Preclinical models have demonstrated that Cd exposure compromises skin immune equilibrium and exacerbates pro-inflammatory cytokine expression, which may underlie its role in promoting chronic dermatoses and premature skin aging (8–10).

A central mechanism in Cd-induced cytotoxicity is the disruption of redox homeostasis via excessive reactive oxygen species (ROS) generation (11). ROS-mediated damage impairs DNA, proteins, and lipids and contributes to mitochondrial dysfunction, a recognized hallmark of aging (6, 12). Mitochondria are not only critical for cellular energy production but also key regulators of redox signaling (13). Within the epidermis, mitochondrial homeostatic collapse is considered a primary driver of aging-related manifestations and the loss of tissue renewal potential (14).

As an NAD^+^-linked deacetylase, Sirtuin 1 (SIRT1) serves as a central orchestrator of mitochondrial homeostasis and the cellular defense mechanisms against oxidative insults (15). SIRT1 modulates the activity of crucial targets such as P53 (16) and superoxide dismutase 2 (SOD2) (17), thereby influencing DNA repair (18), antioxidant defenses (19), and apoptosis (20, 21). Decreased SIRT1 expression has been documented in Cd-exposed tissues and is correlated with exacerbated mitochondrial damage and cell death (19). Restoration of SIRT1 activity, for example via pharmacologic agonists, mitigates Cd-induced oxidative injury and mitochondrial impairment, suggesting a protective role of the SIRT1 axis in environmental toxicology (19, 20, 22).

Despite growing recognition of SIRT1’s role in cellular resilience, its specific involvement in Cd-induced dermal aging remains underexplored. Given the shared molecular hallmarks between systemic aging and Cd toxicity-namely, redox imbalance, mitochondrial damage, and chronic inflammation- we hypothesize that SIRT1 inactivation constitutes a critical node in Cd-driven skin aging.

This study aims to delineate the molecular mechanisms through which Cd accelerates dermal aging, with a particular focus on SIRT1-mediated deacetylation of stress-response proteins and maintenance of redox equilibrium. By employing both *in vitro* dermal fibroblast models and *in vivo* Cd-exposed rats, we seek to establish a mechanistic framework for Cd-induced skin aging and identify SIRT1 as a potential therapeutic target to counteract environmental gerontogens.

## 2 Materials and methods

### 2.1 Experimental reagents and materials

Analytical-grade Cadmium chloride (Cd), nicotinamide adenine dinucleotide (NAD^+^), and other analytical-grade chemicals were supplied by Sigma-Aldrich (St. Louis, MO, USA). For protein quantification, primary antibodies against *β*-actin, acetylated P53 (Lys382), acetylated SOD2 (Lys122), and SIRT1 were sourced from the vendors specified in Table 1. Secondary horseradish peroxidase (HRP)-conjugated antibodies were supplied by Abcam (Cambridge, UK).

**Table 1:** Antibodies and reagents for Western Blotting.

Antibody	Company	Cat. no.	Primary concentration
Ace-SOD2	Abcam	ab214675	1:1000
Ace-P53	Abmart	MN50393	1:1000
ATM	CST	2873	1:1000
Histone H3	Abcam	ab1791	1:1000
P16INK4a	SANTA	sc-1661	1:200
P21CIP1	SANTA	sc-6246	1:200
P53	CST	32532	1:1000
SIRT1	CST	8469	1:1000
β-actin	CST	3700	1:1000
γH2AX	Abcam	ab26350	1:1000

### 2.2 Animal experiments

Adult female Sprague–Dawley rats, weighing 200–220 g (8 weeks of age), were maintained in a controlled environment with ad libitum access to a standard diet and water. Ethical clearance for all experimental protocols was granted by the Institutional Animal Care and Use Committee of Yangzhou University (Permit No. SYXK [Su] 2022–0044). The animals were allocated into either a control group or a Cd-treatment cohort through a randomized process (*n* = 6/group). To induce SIRT1 overexpression, rats in the SIRT1-OVE group received a total volume of 100 μL of AAV-r-SIRT1 (1 × 10^12^ VG/mL) via multi-point intradermal injections into the abdominal skin at the beginning of the third month of the study. Successful overexpression of SIRT1 was validated 1 month post-injection (at the end of the fourth month) via Western blot analysis. Following this confirmation, the Cd-exposed group was allowed free access to drinking water containing Cd (50 mg/L) for 6 months, while the control group received normal drinking water. The AAV-ove-SIRT1 viruses were obtained from Hanheng Biotechnology Co., Ltd.

### 2.3 Cell culture and treatment

The primary rat skin fibroblasts were derived from Pregnant Sprague–Dawley (SD) rats at 18 days gestation. The dorsal and abdominal skin of the fetuses was dissected and minced into small pieces approximately 1 mm × 1 mm in size and transferred into a culture flask. The resulting cell suspension was collected after 2 days and cultured under conditions of 37 °C, 5% CO2, in Dulbecco’s Modified Eagle Medium (DMEM) supplemented with 10% fetal bovine serum (FBS). For the dose–response experiments, primary fibroblasts were seeded in 6-well plates and exposed to a gradient of Cd concentrations (0, 0.2, 0.5, and 1 μM) for 48 h.

Due to the inherent limitations of primary rat skin fibroblasts, such as their low transfection efficiency and limited stability during long-term genetic manipulation, the C3H/10 T1/2, Clone 8 (C3H) cell line was selected for the establishment of the SIRT1-overexpression model. C3H cells are a murine embryonic fibroblast cell line derived from C3H mouse embryos, widely utilized as a model for studying mesenchymal differentiation, toxicological responses, and molecular mechanisms of gene regulation due to their stable growth characteristics and multipotent differentiation potential (23, 24). These cells were cultured in DMEM supplemented with 10% FBS, 100 U/mL penicillin, and 100 μg/mL streptomycin at 37 °C in a humidified atmosphere containing 5% CO₂. For Cd exposure experiments, cells were treated with Cd for 48 h. To determine the optimal concentration for the rescue studies, C3H cells were initially treated with a gradient of Cd concentrations (0, 2, 4, and 6 μM) for 48 h. Based on the assessment of cell viability and senescence markers, 6 μM was identified as the optimal concentration for subsequent SIRT1-overexpression experiments.

### 2.4 SIRT1 overexpression *in vitro*

The C3H/10T1/2, Clone 8 cell line utilized in this study was supplied by Procell Life Science & Technology Co., Ltd. The construction of lentivirus-mediated SIRT1 shRNA was carried out by Shanghai GeneChem. In various experiments, after transduction with lentivirus-mediated SIRT1 shRNA, cells were treated with 6 μM CdCl2 for 48 h, using scramble shRNA as the control.

### 2.5 Detection of cadmium content in skin tissues

Abdominal skin tissues (~0.2 g) were collected, rinsed with distilled water to remove surface contaminants and residual blood, blotted dry, and weighed to obtain wet weight. Samples were digested in a mixed acid solution containing 5 mL nitric acid (HNO₃) and 1 mL perchloric acid (HClO₄). All digestion procedures were performed in a chemical fume hood under strict safety protocols. Predigestion was carried out on a heating plate at 120 °C until the solution became nearly clear, followed by gradual heating to 180 °C until complete digestion was achieved and a colorless, transparent solution was obtained. After cooling to room temperature, the digested samples were diluted to a final volume of 25 mL with deionized water.

Blank solutions were prepared using the same procedure. Cadmium standard solutions were prepared from a certified reference standard (GBW(E)080193) to generate calibration curves. After filtration, Cd concentrations in the samples were measured using a flame atomic absorption spectrophotometer (PinAAcle 900F, PerkinElmer, USA). Calibration curves demonstrated excellent linearity (*R*^2^ > 0.99). Method accuracy was verified using spike recovery tests and standard reference materials. Each sample was analyzed in technical triplicate, and the average value was used for calculation. Cd content was expressed as μg/g wet weight.

### 2.6 Ultrastructural observation via transmission electron microscopy

For the ultrastructural examination of fibroblasts and skin tissues after CdCl_2_ treatment, both were fixed using a consistent method. The images of these samples were then acquired with a HITACHI HT7800 transmission electron microscope.

### 2.7 Senescence-associated *β*-galactosidase (SA-*β*-gal) staining

Assessment of SA-*β*-gal activity was performed utilizing a specific senescence detection kit (Cell Signaling Technology), strictly adhering to the instructions provided by the manufacturer. In brief, cellular specimens were immobilized using 0.5% glutaraldehyde for a 15-min duration at ambient temperature. Following a PBS rinse, the cells were subjected to overnight incubation with the chromogenic substrate at 37 °C. The presence of senescent cells, identified by their distinct blue pigmentation, was quantified across five stochastically selected areas per sample utilizing bright-field microscopy.

### 2.8 Intercellular ROS, total AOC, 8-OHdG and NAD total concentration detection

Intracellular ROS levels were measured using the fluorescent probe 2′,7′-dichlorofluorescin diacetate (DCFH-DA; Sigma-Aldrich). Cells were exposed to 10 μM DCFH-DA (Sigma-Aldrich) for 30 min at 37 °C in a light-shielded environment to label endogenous ROS. After removing excess probe with PBS washes, the fluorescent emission was monitored using an Olympus microscope. Quantitative analysis of the fluorescence intensity was subsequently performed through ImageJ software to assess redox status.

The Total Antioxidant Capacity (T-AOC) was assessed by homogenizing cells, obtaining supernatant through centrifugation, and mixing it with a T-AOC reagent (Beyotime, Shanghai, China). Specimens were maintained at approximately 25 °C for 6 min to facilitate the reaction. Absorbance was measured at 405 nm by microplate reader (BioTek Synergy HTX). T-AOC levels were then determined by comparison with a standard curve (T-AOC content / Protein mass).

Cells and skin tissue were homogenated in PBS (PH7.4), then centrifugation at 5000 g for 5 min at 4 °C. Supernatant was used to measure 8-Hydroxy-desoxyguanosine (8-OHdG) by ELISA kits (Mibo China). The optical density of each well was measured at 450 nm.

According to the protocol provided by Beyotime (#S0175), the quantification of total NAD involved an initial sample preparation where 1 × 10^6^ cells were spun down at 1000 g (4 °C) for 5 min. The resulting pellet was resuspended in 200 μL of ice-cold NAD+/NADH extraction solution after discarding the culture medium. Concurrently, 20 mg of skin tissue fragments were prepared in 400 μL of extraction buffer. Both cell and tissue lysates were then vortexed and cleared by centrifugation at 12,000 *g* for 10 min at 4 °C. The cleared supernatant was subsequently subjected to colorimetric analysis at 450 nm using a 96-well plate reader.

### 2.9 Mitochondrial membrane potential (ΔΨm) assessment

Mitochondrial membrane potential was determined via the JC-1 Mitochondrial Membrane Potential Assay Kit (Beyotime, China). Upon completing the 37 °C incubation (20 min) with the staining solution, specimens were cleansed and subjected to fluorescent imaging. The ΔΨm status was subsequently derived from the fluorescence intensity ratio of red J-aggregates to green J-monomers.

### 2.10 Western blot analysis

Cellular and tissue proteins were isolated utilizing RIPA lysis buffer supplemented with a cocktail of phosphatase and protease inhibitors (Beyotime). After quantifying protein concentrations via a BCA assay (Thermo Fisher Scientific), 30 μg of each protein sample was resolved using SDS-PAGE and subsequently electroblotted onto PVDF membranes (Millipore). The membranes were then subjected to a 1-h blocking phase in 5% skim milk at ambient temperature, followed by an overnight primary antibody incubation at 4 °C. Post-washing, HRP-linked secondary antibodies were applied for 1 h. Finally, immunoreactive bands were developed using an ECL chemiluminescence system (Bio-Rad) and the signal intensities were analyzed through ImageJ.

### 2.11 Cell viability measurement

Primary rat dermal fibroblasts were inoculated into E-Plates at a seeding density of 5 × 10^3^ cells/well. The assembly was then integrated into the Real-Time Cellular Analysis (RTCA) platform (Roche, Mannheim, Germany) within a CO₂ incubator (37 °C, 5%). The system automatically logged the Cell Index (CI)— a composite parameter of cell adhesion, morphology, and population—at 15-min frequencies. Upon reaching the logarithmic growth stage, the cultures were challenged with varying Cd concentrations. For comparative accuracy, the CI was normalized to a baseline of 1.0 at the onset of treatment, with subsequent proliferation kinetics tracked continuously for 48 h following Cd treatment.

### 2.12 Cell cycle assay

The primary rat skin fibroblasts were immobilized in 70% ethanol for a 12-h duration. Subsequent to fixation, the cells were subjected to a 15-min staining process with propidium iodide (PI)/RNase buffer (Beyotime, C1052) under light-shielded conditions. The fluorescent profiles were then acquired utilizing a flow cytometer (LSRFortessa, BD Biosciences, USA).

### 2.13 TUNEL staining

The primary rat skin fibroblasts were immobilized in 4% paraformaldehyde, followed by permeabilization utilizing 0.1% Triton X-100. The specimens were then subjected to a 1-h incubation with the TUNEL reaction cocktail (Beyotime, China) at 37 °C in a light-shielded environment. Finally, apoptotic signals were captured using a Nikon fluorescence microscope.

### 2.14 Statistical analysis

Quantitative results are expressed as the mean ± standard error of the mean (SEM), derived from a minimum of three separate replicates. GraphPad Prism 8.0 (GraphPad Software, San Diego, CA, USA) was employed to conduct all statistical evaluations. To determine differences between two specific groups, the unpaired Student’s *t*-test was utilized. For assessments involving more than two cohorts, one-way analysis of variance (ANOVA) followed by Tukey’s multiple comparison test was applied. Statistical significance was predefined as a *p*-value less than 0.05.

## 3 Results

### 3.1 Cd exposure induces senescence in dermal fibroblasts

**Figure 1:**
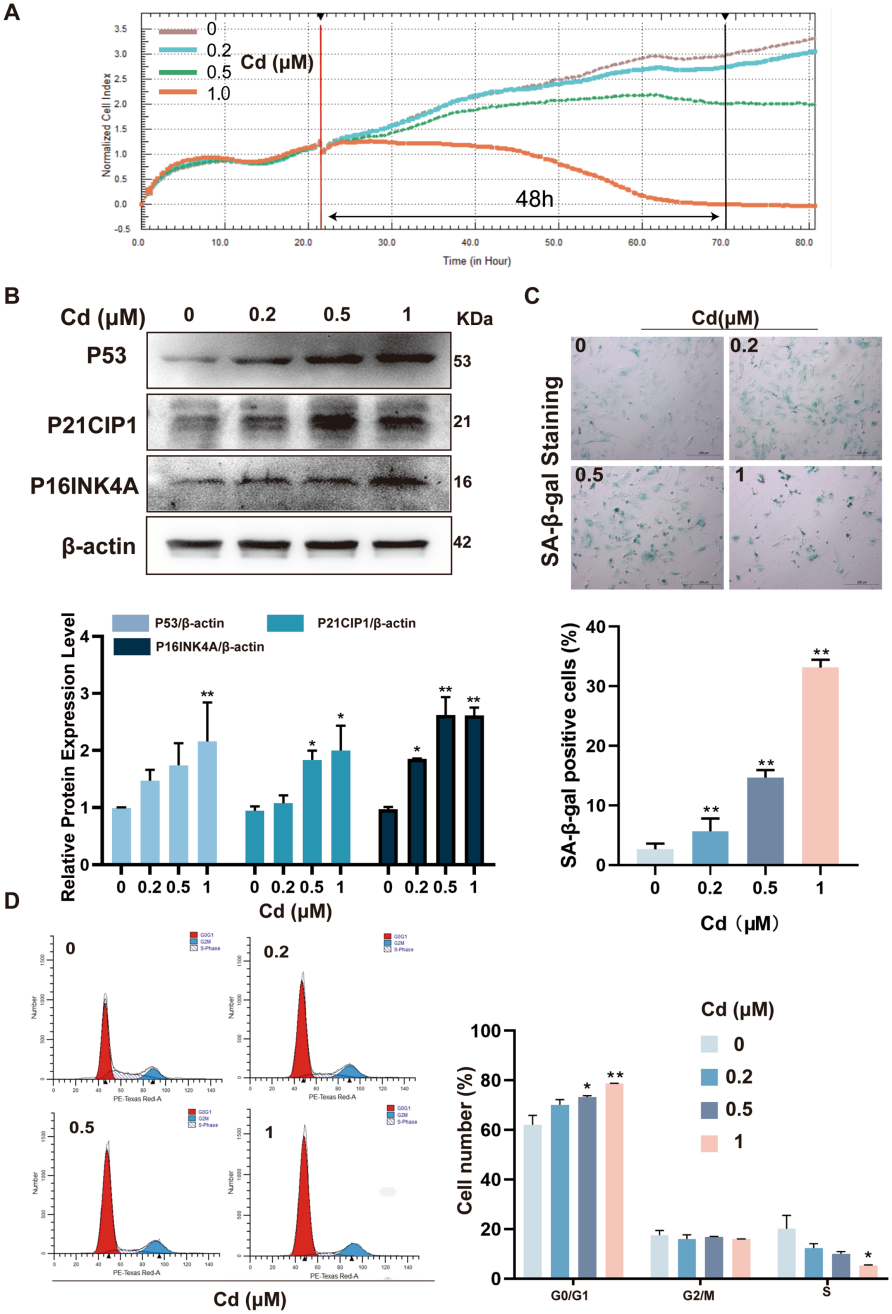
Exposure to CdCl_2_ prompts an age-simulating phenotype in rat dermal fibroblasts. **(A)** Cell proliferation was monitored using a real-time cell analysis (RTCA) system following exposure to increasing concentrations of Cd (0, 0.2, 0.5, and 1 μM) for up to 48 h. **(B)** Western blot analysis of senescence-associated markers P53, P21CIP1, and P16INK4A. **(C)** SA-β-gal staining showing increased senescent cell burden. **(D)** Cell cycle distribution determined by flow cytometry, highlighting G1/G0 phase arrest. All experiments were performed 48 h post Cd exposure and were repeated independently three times (biological replicates). Independent experiments were performed in triplicate. Quantitative results are expressed as the mean ± SD. *p < 0.05, **p < 0.01 *vs.* control group.

To investigate the cellular effects of Cd on skin aging, we established an *in vitro* model using primary rat dermal fibroblasts. Cells were exposed to a gradient of Cd concentrations (0–1 μM) for 48 h. Cd treatment resulted in a dose-dependent decline in cell proliferation, with a significant reduction observed at 1 μM (Figure 1A), indicating substantial cytotoxic stress at this threshold. Morphologically, Cd-exposed fibroblasts exhibited decreased adhesion and spreading ability, along with an overall reduction in cell viability at higher Cd concentrations, reflecting impaired cellular attachment and survival under Cd-induced stress.

We next examined cellular senescence by analyzing the expression of classical senescence-associated markers. Western blotting revealed that exposure to Cd markedly upregulated the protein levels of P53, P21CIP1, and P16INK4A, key regulators of cell cycle arrest and senescence (Figure 1B) (25, 26). Consistent with the aforementioned observations, SA-*β*-gal histochemical staining revealed a marked elevation in the density of blue-pigmented senescent cells within the Cd-exposed cohorts relative to the control group. It is worth noting that while elevated Cd concentrations led to a reduction in total cell density and viability, the percentage of SA-β-gal-positive individuals among the surviving cells exhibited a substantial increase (Figure 1C) (27). Supporting these findings, flow cytometric profiling indicated that Cd exposure triggered a prominent G1/G0 phase arrest (Figure 1D), a hallmark characteristic of the senescence-associated growth cessation phenotype.

Collectively, these results establish that Cd exposure induces a robust senescent phenotype in dermal fibroblasts, characterized by impaired proliferation, upregulation of senescence markers, and cell cycle arrest. This model effectively recapitulates key molecular features of heavy metal–induced dermal aging and provides a foundation for further mechanistic investigation.

### 3.2 Cd triggers oxidative stress and DNA damage

**Figure 2:**
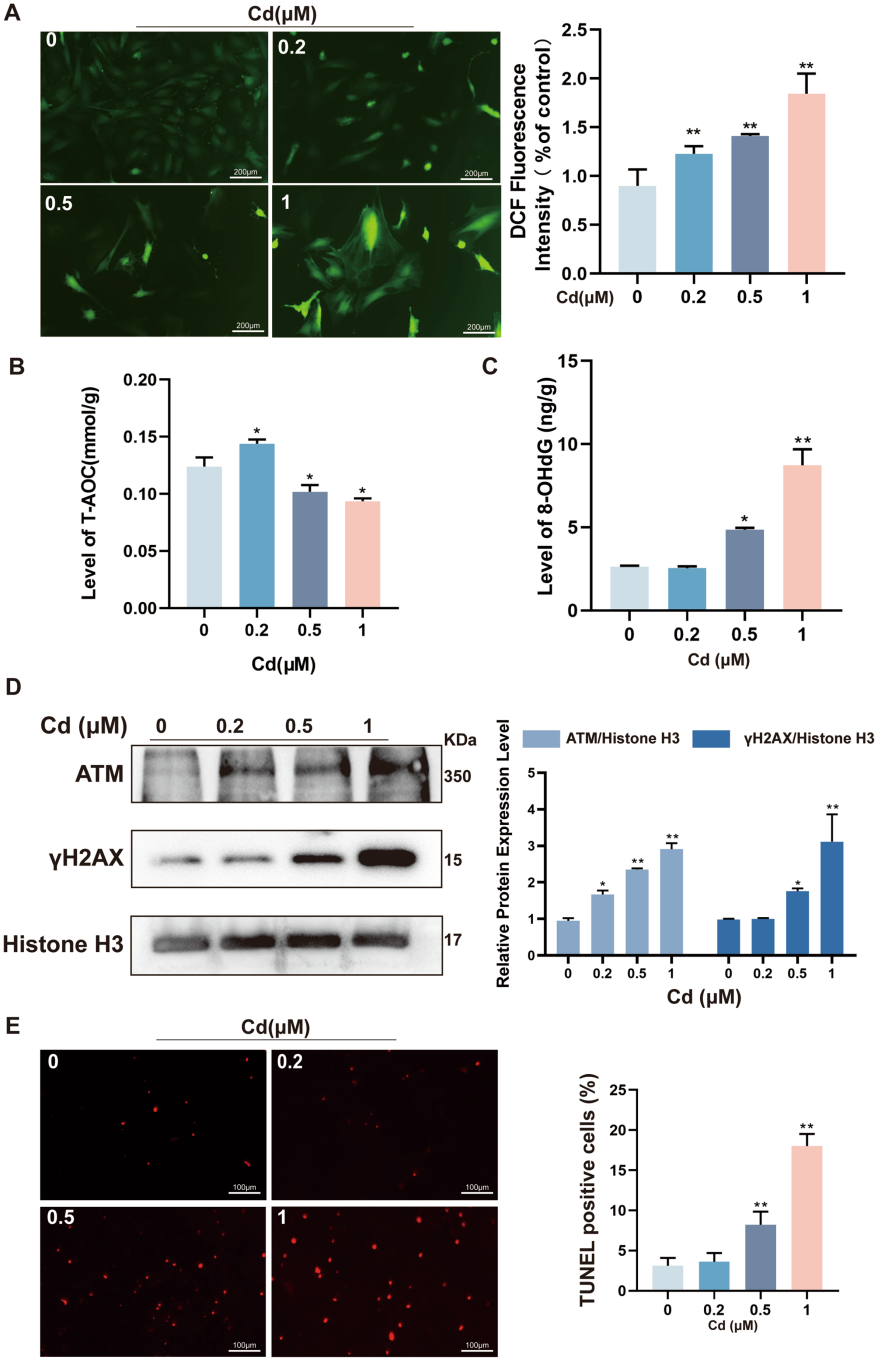
Cd induces oxidative stress and DNA damage in rat dermal fibroblasts. **(A)** ROS accumulation assessed by DCFH-DA staining. **(B)** Total antioxidant capacity (AOC) measured via spectrophotometric assay. **(C)** Quantification of oxidative DNA damage via 8-OHdG levels. **(D)** Western blot analysis of DNA damage response proteins ATM and γH2AX. **(E)** TUNEL staining for apoptotic nuclear fragmentation. All experiments were performed 48 h post Cd exposure and were repeated independently three times (biological replicates). Independent experiments were performed in triplicate. Quantitative results are expressed as the mean ± SD. *p < 0.05, **p < 0.01 *vs.* control group.

To elucidate the mechanisms underlying Cd-induced cellular senescence, we examined markers of oxidative stress and genotoxic damage. Fluorescent imaging using DCFH-DA staining demonstrated a significant elevation of intracellular reactive oxygen species (ROS) levels following Cd exposure, indicating heightened oxidative stress in treated fibroblasts (Figure 2A). This oxidative derangement was further substantiated by a prominent decline in total antioxidant capacity (AOC), alongside a simultaneous elevation of 8-hydroxy-2′-deoxyguanosine (8-OHdG), which serves as a classic indicator of oxidative-mediated DNA lesions (Figures 2B,C). Western blot analysis revealed upregulation of key DNA damage response proteins, including ATM and γH2AX, in Cd-treated cells (Figure 2D). Direct evidence of apoptosis and DNA damage was provided by TUNEL analysis, where Cd-treated fibroblasts exhibited pronounced nuclear fragmentation and apoptotic morphology (Figure 2E).

Taken together, these results demonstrate that Cd induces profound oxidative stress and DNA damage in dermal fibroblasts, contributing to cellular dysfunction and potentially promoting senescence.

### 3.3 Cd induces mitochondrial dysfunction and SIRT1 downregulation

**Figure 3:**
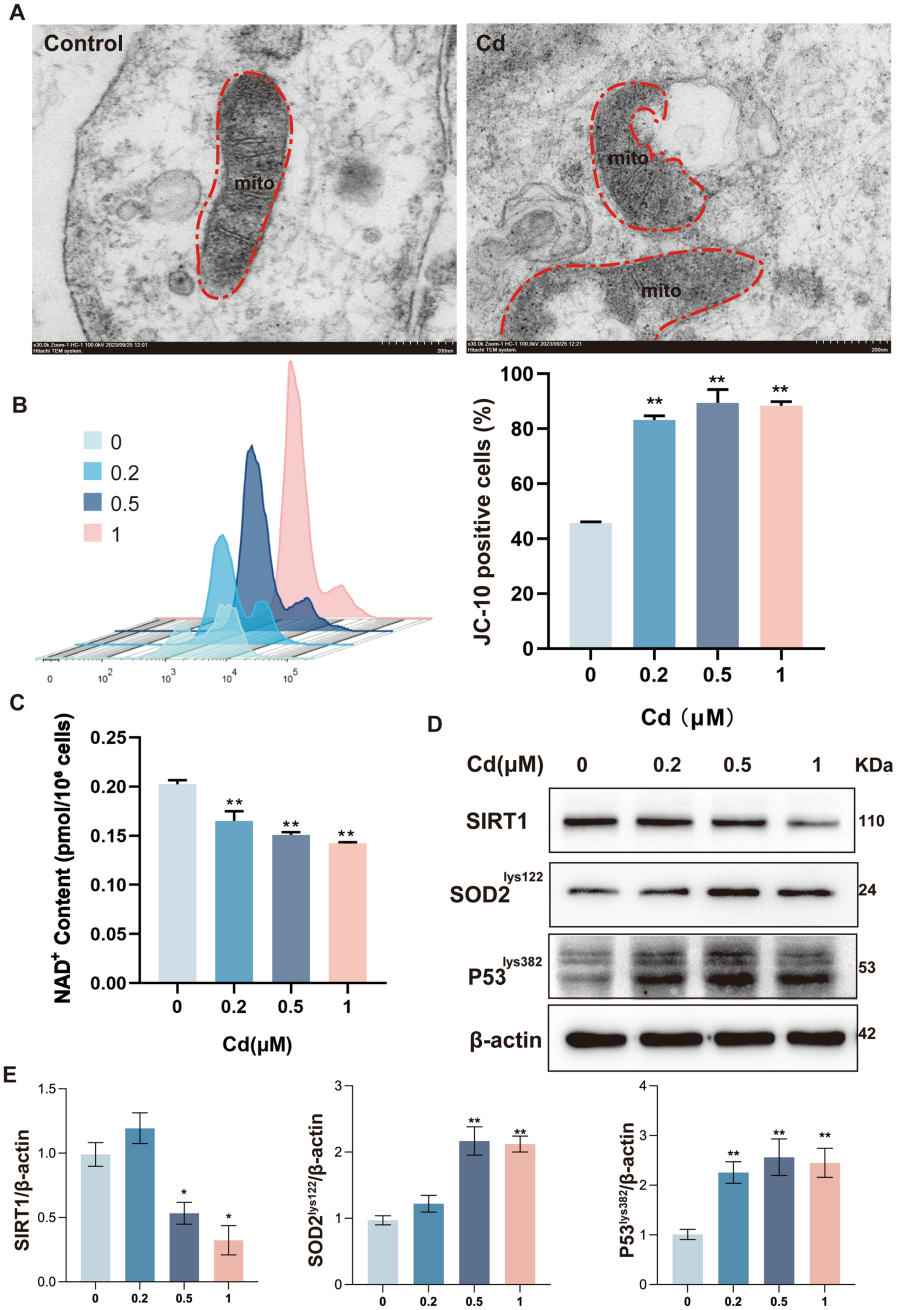
Cd impairs mitochondrial integrity and suppresses SIRT1 activity. **(A)** Transmission electron microscopy (TEM) images showing mitochondrial swelling and cristae loss. **(B)** JC-10 staining reveals disrupted mitochondrial membrane potential (ΔΨm). **(C)** NAD^+^ content determined by ELISA. **(D)** Western blot showing decreased SIRT1 expression and increased acetylation of SOD2 (Lys122) and P53 (Lys382). All experiments were performed 48 h post Cd exposure and were repeated independently three times (biological replicates). Independent experiments were performed in triplicate. **(E)** Densitometric analysis of SIRT1, acetylated SOD2 (Lys122), and acetylated P53 (Lys382) protein levels normalized to *β*-actin. Quantitative results are expressed as the mean ± SD. *p < 0.05, **p < 0.01 vs. control group.

To further investigate the intracellular consequences of Cd exposure, we examined mitochondrial ultrastructure and function in dermal fibroblasts. Ultrastructural analysis via transmission electron microscopy (TEM) pronounced mitochondrial abnormalities, including swelling, cristae disruption, and loss of matrix density, indicative of structural mitochondrial damage (Figure 3A). Functional impairment was corroborated by JC-10 staining, which demonstrated a significant reduction in ΔΨm in Cd-treated cells compared to controls (Figure 3B), suggesting mitochondrial depolarization and dysfunction. In parallel, ELISA quantification of cellular NAD^+^ levels revealed a marked depletion following Cd exposure (Figure 3C), implicating energetic stress and potential inhibition of NAD^+^-dependent enzymes.

The enzymatic status of SIRT1 and the integrity of its downstream signaling pathways were investigated, predicated on its biological identity as an NAD^+^-reliant protein deacetylase. Western blot analysis showed a significant decrease in SIRT1 protein expression, along with hyperacetylation of its known substrates P53 (at Lys382) and SOD2 (at Lys122), consistent with impaired SIRT1 enzymatic function (Figures 3D,E). Collectively, these findings suggest that Cd exposure disrupts mitochondrial structure and function, depletes cellular NAD^+^ levels, and suppresses SIRT1-mediated protective signaling, thereby amplifying oxidative and senescence-related cellular damage.

### 3.4 SIRT1 overexpression mitigates cd-induced senescence and DNA damage

Notably, our initial experiments revealed that the C3H immortalized cell line exhibited a significantly higher tolerance to Cd toxicity compared to primary rat fibroblasts. To account for this difference in cellular sensitivity and to ensure a reproducible senescence model, we expanded the testing range for C3H cells. Specifically, treatment with increasing concentrations of Cd (0, 2, 4, 6 μM) progressively elevated the expression of senescence-associated proteins and increased SA-*β*-gal positivity, with 6 μM showing the most pronounced effects (Supplementary Figures S1A,B). Based on our preliminary dose–response experiments, 6 μM Cd was selected as an optimal concentration for subsequent mechanistic studies, as it induced a robust senescent phenotype in C3H cells while maintaining sufficient cell viability for downstream analyses.

**Figure 4:**
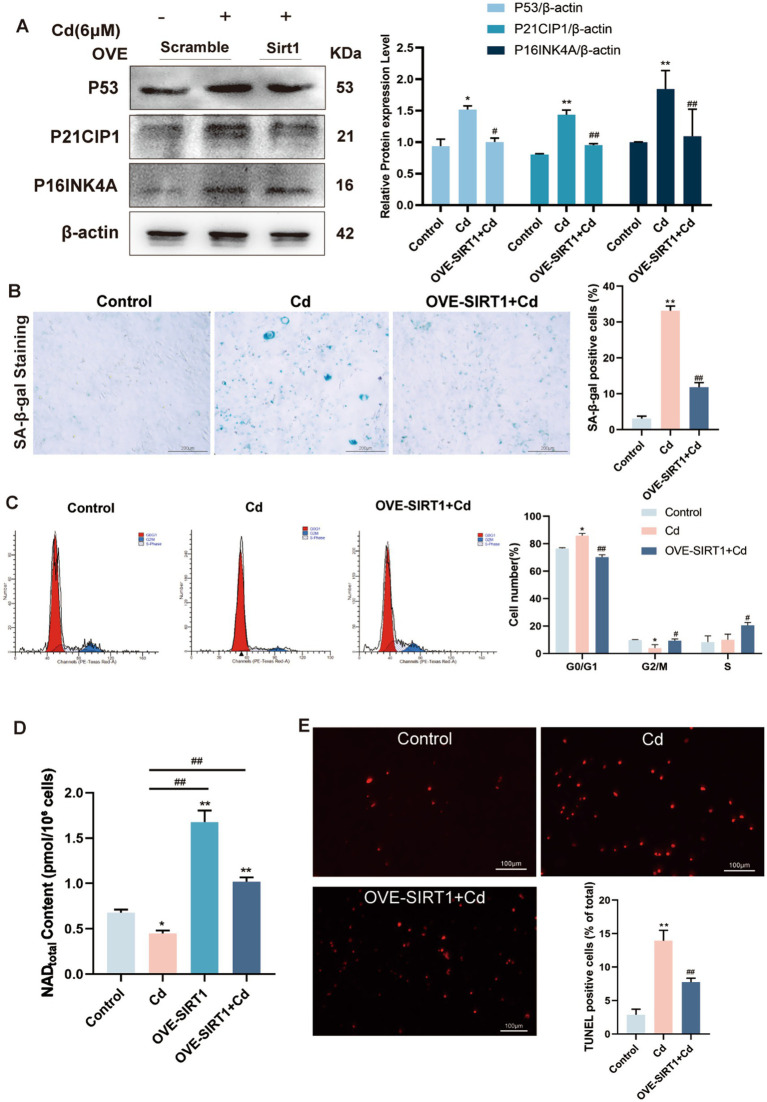
Overexpressing SIRT1 in C3H lessens the aging effects caused by CdCl_2_ exposure. **(A)** Western blot of P53, P21CIP1, and P16INK4A in fibroblasts with or without SIRT1 overexpression. **(B)** SA-β-gal staining of senescent cells. **(C)** Quantification of G1/G0 cell cycle arrest via flow cytometry. **(D)** Intracellular NAD^+^ levels measured by ELISA. **(E)** TUNEL staining showing reduced apoptotic nuclei in SIRT1-overexpressing cells. All experiments were performed 48 h post Cd exposure and were repeated independently three times (biological replicates). Independent experiments were performed in triplicate. Quantitative results are expressed as the mean ± SD. *p < 0.05, **p < 0.01 vs. control; ^#^*p* < 0.05, ^##^*p* < 0.01 *vs.* Cd group.

To determine whether SIRT1 plays a protective role against Cd-induced cellular stress, we overexpressed SIRT1 in C3H cells via lentiviral vector and subsequently exposed the cells to 6 μM Cd. Compared with negative control, SIRT1-overexpressing fibroblasts exhibited markedly reduced susceptibility to Cd-induced senescence and apoptosis. Specifically, Western blot analysis revealed decreased expression of senescence-associated proteins P53, P21CIP1, and P16INK4A in the SIRT1-overexpressing group (Figure 4A). Consistent with these molecular findings, SA-β-gal staining showed a significant reduction in senescent cell burden (Figure 4B), and flow cytometric analysis demonstrated a lower proportion of cells arrested in the G1/G0 phase (Figure 4C), indicating partial restoration of cell cycle progression. Furthermore, overexpression of SIRT1 significantly rescued intracellular NAD^+^ levels (Figure 4D), which are otherwise depleted by Cd treatment, supporting the functional activity of the NAD^+^-dependent deacetylase. TUNEL assays also confirmed a notable decrease in apoptotic nuclear fragmentation among SIRT1-overexpressing cells compared to Cd-treated controls (Figure 4E).

Taken together, these observations confirm that SIRT1 exerts a multifaceted protective effect against Cd-induced injury by quenching reactive species, upholding cell cycle homeostasis, and diminishing the apoptotic burden in primary fibroblasts.

### 3.5 SIRT1 suppresses cd-induced ROS and Restores mitochondrial function

**Figure 5:**
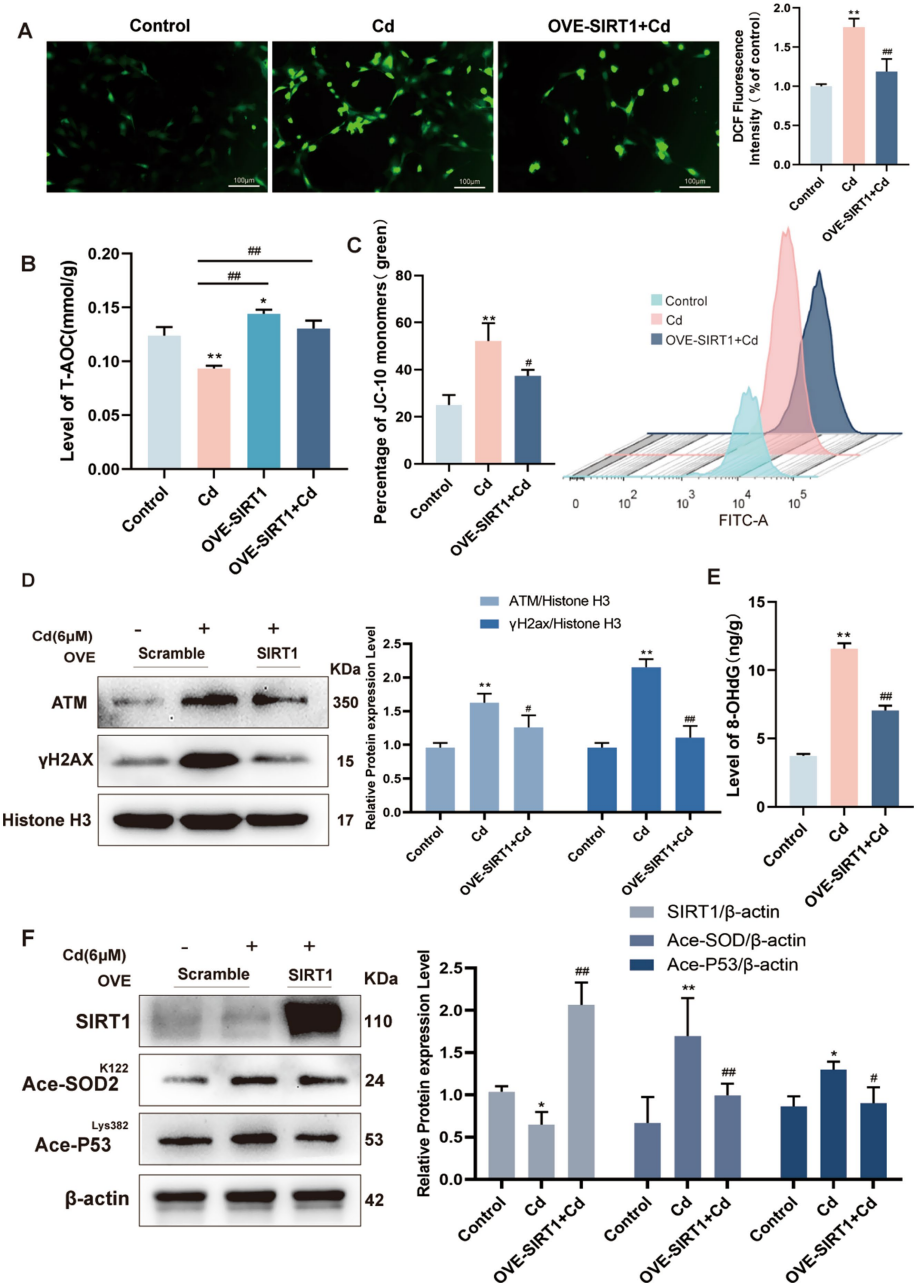
SIRT1 restores redox balance, mitochondrial function, and genomic stability under Cd stress. **(A)** Intracellular ROS detected by DCFH-DA staining. **(B)** Measurement of total antioxidant capacity (AOC). **(C)** JC-10 staining reveals recovery of ΔΨm in SIRT1-overexpressing fibroblasts. **(D)** Western blot of γH2AX and ATM. **(E)** 8-OHdG levels as a marker of oxidative DNA damage. **(F)** Reversal of P53 and SOD2 acetylation upon SIRT1 overexpression. All experiments were performed 48 h post Cd exposure and were repeated independently three times (biological replicates). Independent experiments were performed in triplicate. Quantitative results are expressed as the mean ± SD. *p < 0.05, **p < 0.01 *vs.* control; ^#^*p* < 0.05, ^##^*p* < 0.01 *vs.* Cd group.

To delve into the protective molecular network of SIRT1, we evaluated its impact on oxidative stress, mitochondrial function, and DNA integrity in Cd-exposed fibroblasts. Overexpression of SIRT1 significantly suppressed the accumulation of ROS, as demonstrated by reduced DCFH-DA fluorescence intensity (Figure 5A). This attenuation of oxidative stress was accompanied by normalization of T-AOC, suggesting restored redox homeostasis (Figure 5B). In addition, JC-10 staining revealed a substantial recovery of ΔΨm in SIRT1-overexpressing cells, indicating preserved mitochondrial function under Cd stress (Figure 5C).

At the genomic level, SIRT1 overexpression led to marked reductions in the expression of DNA damage response proteins, including γH2AX and ATM (Figure 5D). Levels of 8-OHdG, a sensitive marker of oxidative DNA damage, were also significantly decreased (Figure 5E). Western blot analysis further demonstrated that SIRT1 overexpression reversed Cd-induced hyperacetylation of SOD2 and P53 (Figure 5F), indicating restored SIRT1 enzymatic activity and downstream regulation. Collectively, these findings confirm that SIRT1 confers substantial protection against Cd-induced mitochondrial dysfunction and DNA damage by modulating redox balance, maintaining mitochondrial integrity, and promoting genomic stability.

### 3.6 . SIRT1 overexpression protects against cd-induced skin aging *in vivo*

**Figure 6:**
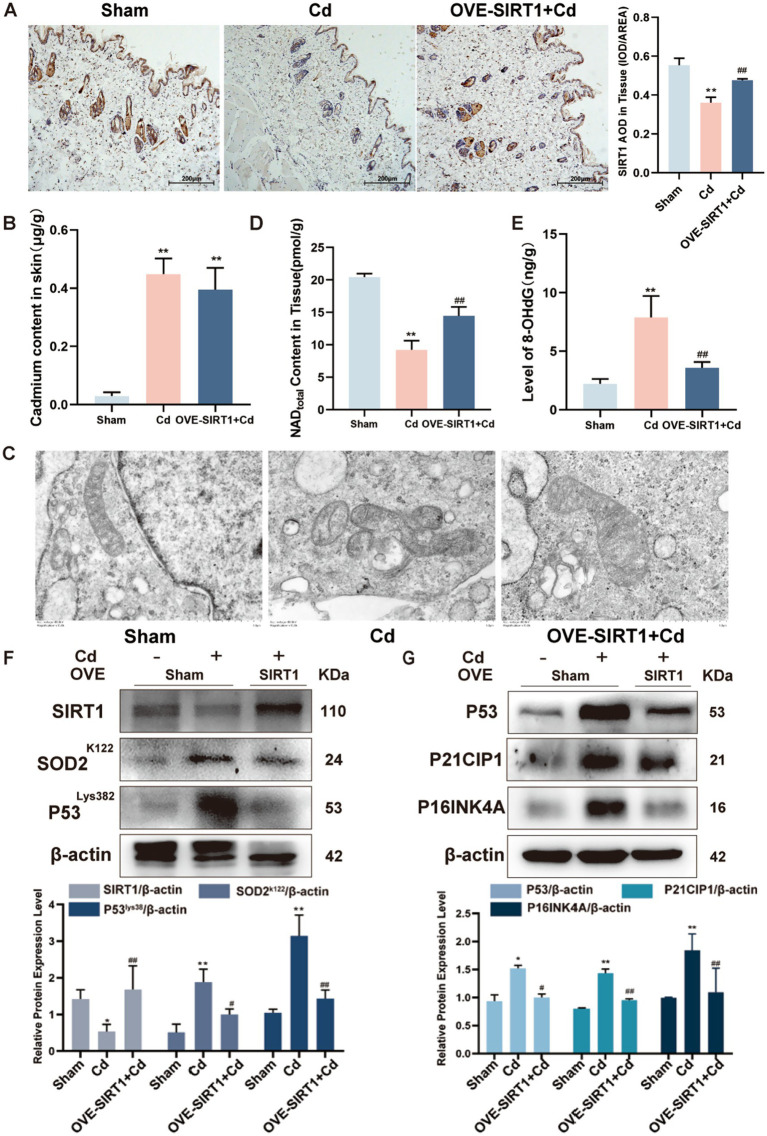
SIRT1 overexpression alleviates Cd-induced skin aging *in vivo*. **(A)** Immunohistochemistry confirming SIRT1 upregulation in rat skin tissue. **(B)** Cd accumulation in skin measured by atomic absorption spectroscopy. **(C)** TEM images showing preserved mitochondrial structure in SIRT1-treated rats. **(D)** Skin NAD^+^ levels measured by ELISA. **(E)** 8-OHdG content reflecting oxidative DNA damage. **(F,G)** Western blot analysis of senescence markers P53, P21CIP1, and P16INK4A. Independent experiments were performed in triplicate. Quantitative results are expressed as the mean ± SD. *p < 0.05, **p < 0.01 vs. sham; ^#^*p* < 0.05, ^##^*p* < 0.01 *vs.* Cd group.

To validate the protective effects of SIRT1 *in vivo*, we employed a chronic Cd exposure rat model combined with skin-targeted SIRT1 overexpression. Immunohistochemical staining confirmed successful upregulation of SIRT1 protein in the skin tissues of AAV-*SIRT1*–treated rats (Figure 6A). Atomic absorption spectroscopy revealed significantly lower Cd accumulation in the skin of the SIRT1-overexpressing group versus the group treated with Cd alone (Figure 6B), suggesting a potential enhancement of detoxification or metal efflux mechanisms. TEM further showed preserved mitochondrial morphology in SIRT1-overexpressing skin, with intact cristae and reduced swelling relative to Cd-treated controls (Figure 6C). Biochemical assays demonstrated that SIRT1 overexpression restored NAD^+^ content (Figure 6D) and significantly reduced 8-OHdG levels in skin tissue, indicating diminished oxidative DNA damage (Figure 6E). Consistent with a reduction in cellular senescence, Western blot results indicated that the protein abundance of P53, P21CIP1, and P16INK4A was lower (Figures 6F,G), confirming the mitigation of senescence-associated pathways.

These *in vivo* results reinforce the protective role of SIRT1 in mitigating Cd-induced dermal aging, highlighting its ability to preserve mitochondrial integrity, reduce oxidative stress, and suppress senescence pathways.

## 4 Discussion

Heavy metals such as Cd represent an underrecognized class of environmental gerontogens, agents that accelerate biological aging by disrupting redox balance, genomic stability, and cellular homeostasis (28, 29). While Cd’s systemic toxicity has been extensively studied in renal (30), hepatic (31), and neural tissues (11), its role in cutaneous aging is only recently gaining attention. The present study provides mechanistic evidence that Cd, a widespread environmental and dietary contaminant, accelerates skin aging through oxidative stress–driven DNA damage and mitochondrial dysfunction. Cd has been shown to induce oxidative stress via Fenton-like reactions, inhibition of antioxidant enzymes, and interference with mitochondrial electron transport chains (32). In skin, where oxidative balance is vital to structural integrity and barrier function, chronic Cd exposure may disproportionately impair fibroblast and stem cell populations (33).

Cd exposure has been widely implicated in multiple organ toxicities; however, its impact on skin aging has been comparatively underexplored (34). Our findings show that low-dose chronic Cd exposure induces hallmark features of cellular aging in dermal fibroblasts, including elevated expression of senescence markers (P53, P21CIP1, P16INK4A), increased SA-*β*-gal positivity, and G1/G0 cell cycle arrest. These effects are consistent with prior studies implicating heavy metals as environmental gerontogens that activate stress-response pathways and promote cellular senescence through oxidative injury (35–37). Consistent with recent reports linking Cd accumulation in skin to altered collagen synthesis, lipid peroxidation, and impaired wound healing (38–41). We observed that Cd exposure robustly increased intracellular ROS levels and activated canonical DNA damage responses. Mechanistically, we observed that Cd-induced ROS accumulation disrupts redox balance, reduces antioxidant capacity, and activates DNA damage responses, as evidenced by upregulated γH2AX and ATM expression and increased 8-OHdG levels. These findings align with established roles of ROS in mitochondrial injury and aging pathogenesis (37, 42, 43).

Given the central role of mitochondria as both a source and target of ROS, we next examined mitochondrial integrity. Functional assays demonstrated that Cd exposure compromised mitochondrial membrane potential, reduced NAD^+^ availability, and increased intracellular ROS levels. Importantly, transmission electron microscopy (TEM) provided direct ultrastructural evidence of mitochondrial injury. Cd-treated dermal fibroblasts exhibited pronounced mitochondrial swelling, disrupted and fragmented cristae, vacuolization, and electron-dense inclusions, hallmark features of mitochondrial stress and degeneration. Such structural abnormalities are widely recognized as early indicators of mitochondrial dysfunction and are closely associated with impaired oxidative phosphorylation efficiency and excessive ROS generation (44, 45). These ultrastructural abnormalities are highly consistent with our functional observations, including loss of mitochondrial membrane potential, reduced NAD^+^ availability, and elevated intracellular ROS levels. Previous studies have demonstrated that cadmium targets mitochondria as a key intracellular site of toxicity, where it disrupts electron transport chain complexes and mitochondrial membrane integrity, leading to impaired oxidative phosphorylation and enhanced ROS production (46, 47).

In line with this mechanism, our data indicate that Cd-induced mitochondrial dysfunction is accompanied by persistent activation of the DNA damage response, as evidenced by increased γH2AX accumulation and ATM activation, both of which are well-established markers of DNA double-strand break signaling and genomic instability (48). Mitochondrial structural disruption provides a plausible mechanistic basis for these observations, as cadmium exposure is known to impair mitochondrial function and promote excessive production of reactive oxygen species (ROS). These mitochondrial ROS can diffuse into the nucleus and induce oxidative DNA damage, thereby triggering sustained activation of ATM-dependent DNA damage signaling pathways (49). Consistent with this model, robust upregulation of γH2AX and ATM was detected in our study, suggesting prolonged DNA damage signaling rather than a transient oxidative stress response. Similar associations between Cd-induced mitochondrial dysfunction, ROS accumulation, and activation of DNA damage responses have been reported in multiple cell types and tissues following cadmium exposure (50, 51).

At the regulatory level, our data identify the NAD^+^-dependent deacetylase SIRT1 as a critical node linking Cd-induced mitochondrial dysfunction to cellular senescence. Cd exposure markedly reduced intracellular NAD^+^ levels and suppressed SIRT1 expression, resulting in hyperacetylation of P53 and SOD2. And we found that genetic overexpression of SIRT1 effectively reversed these effects, significantly attenuating the hyperacetylation of P53 and SOD2, thereby restoring their functional roles in cellular homeostasis. These findings are consistent with previous studies in neuronal and renal models, where Cd-mediated inhibition of SIRT1 exacerbated mitochondrial injury and oxidative stress (19, 52).

Importantly, SIRT1 is more than a redox regulator. It modulates chromatin remodeling (53), autophagy (54), circadian rhythm (55), and the DNA damage response (DDR) (56) functions that position it as a nodal point between environmental stress and aging phenotypes (57–59). In skin, SIRT1 activation has been shown to enhance dermal fibroblast proliferation, protect against UV-induced apoptosis, and promote matrix remodeling via deacetylation of FOXO, P53, and NF-κB pathways (60, 61). Our data expand this paradigm to include Cd-induced aging, demonstrating that genetic overexpression of SIRT1 restores redox balance, reduces 8-OHdG levels, preserves mitochondrial morphology, and attenuates senescence markers *in vivo*.

Notably, our data reveal that SIRT1 overexpression also markedly preserved mitochondrial ultrastructure, as evidenced by restored cristae organization and reduced mitochondrial swelling *in vivo*, suggesting that SIRT1 safeguards mitochondrial integrity not only at the biochemical level but also at the structural level. This protective effect is likely mediated through improved redox homeostasis, enhanced mitochondrial quality control, and deacetylation-dependent regulation of antioxidant defenses such as SOD2. Collectively, these findings position mitochondrial ultrastructural damage as a critical morphological correlate of Cd-induced oxidative stress, DNA damage, and cellular senescence in the skin.

Interestingly, recent studies have explored the epigenetic plasticity of aging, showing that environmental toxins like Cd can alter histone acetylation, DNA methylation, and miRNA profiles long before phenotypic senescence appears (62, 63). In this context, SIRT1 serves as a sensor and effector of metabolic and epigenetic states, linking nutrient availability (via NAD^+^) to transcriptional responses. Another important implication lies in therapeutic modulation. Pharmacological activators of SIRT1, including resveratrol, nicotinamide riboside (NR), and SRT1720, have shown promise in models of neurodegeneration, metabolic syndrome, and radiation-induced damage (64, 65). Whether these agents can reverse or prevent Cd-mediated skin aging remains to be seen, but our study lays the groundwork for such exploration. That said, our findings should be interpreted with caution. The study focused on female rats and acute exposures; sex-specific responses, cumulative lifelong exposure, and interactions with other pollutants (e.g., arsenic, lead) were not examined. Additionally, while SIRT1 overexpression was effective in our model, a systems biology approach integrating multiple pathways (e.g., Nrf2, AMPK, mTOR) would better reflect the complexity of environmental aging.

Our study reinforces the concept of cadmium as a dermal gerontogen, mechanistically linking its effects to mitochondrial dysfunction, oxidative DNA damage, and epigenetic regulation via SIRT1. This finding resonates with an expanding literature that redefines the skin as more than a rudimentary physical shield; instead, it is increasingly recognized as a dynamic interface and a sentinel of systemic aging triggered by environmental stressors. Targeting SIRT1 or its upstream NAD^+^ axis may offer a promising intervention point, not only for mitigating Cd toxicity, but also for addressing broader age-related dermal degeneration.

However, several limitations in the current study warrant further investigation. While we observed mitochondrial impairment, the specific impact of Cd on mitochondrial dynamics—such as the balance between fission, fusion, and mitophagy—remains to be elucidated. Furthermore, although SIRT1 is a known modulator of the Senescence-Associated Secretory Phenotype (SASP), our current data does not account for the paracrine signaling effects of Cd-induced senescent cells on the dermal microenvironment. Additionally, while SIRT1 activity appears to mitigate Cd-induced damage, the precise mechanisms by which it limits or promotes the clearance of Cd accumulation in skin tissue were not explored in this study.

Future work should explore how Cd exposure affects other sirtuins, PARP family members, and DNA methylation pathways involved in cutaneous aging. Additionally, pharmacological modulation of SIRT1 using clinically available activators such as resveratrol or NAD^+^ precursors could be evaluated for translational potential.

## Data Availability

The raw data supporting the conclusions of this article will be made available by the authors, without undue reservation.

## Supplementary material

SUPPLEMENTARY FIGURE S1 Effects of Cd on senescence induction in C3H cells and validation of SIRT1 overexpression *in vivo*. **(A)** Western blot analysis of senescence-associated proteins (including SIRT1, P53, P21CIP1, and P16INK4A) in C3H cells exposed to increasing concentrations of Cd (0, 2, 4, and 6 μM). **(B)** Senescence-associated β-galactosidase (SA-beta-gal) staining demonstrating an increased proportion of senescent cells with rising Cd concentrations (0, 2, 4, and 6 μM). Scale bar = 200 μm. **(C)** Validation of SIRT1 overexpression in the skin tissues of Sprague-Dawley rats following AAV-r-SIRT1 administration, confirming the successful upregulation of SIRT1 protein levels *in vivo*. Independent experiments were performed in triplicate. Quantitative results are expressed as the mean ± SD. **P* < 0.05, ***P* < 0.01 vs. control; #*P* < 0.05, ##*P* < 0.01 vs. Cd group.

## Glossary

8-OHdG: 8-Hydroxy-2′-deoxyguanosine
AAV: Adeno-associated virus
ATM: Ataxia-telangiectasia mutated
BCA: Bicinchoninic acid
Cd: Cadmium
CI: Cell Index
DMEM: Dulbecco’s Modified Eagle Medium
ELISA: Enzyme-linked immunosorbent assay
FBS: Fetal bovine serum
ΔΨm: Mitochondrial membrane potential
NAD^+^: Nicotinamide adenine dinucleotide
RIPA: Radioimmunoprecipitation assay buffer
ROS: Reactive oxygen species
RTCA: Real-Time Cellular Analysis
SA-β-gal: Senescence-associated β-galactosidase
SD rats: Sprague–Dawley rats
SEM: Standard error of the mean
SIRT1: Sirtuin 1
SOD2: Superoxide dismutase 2
T-AOC: Total Antioxidant Capacity
TEM: Transmission electron microscopy
TUNEL: Terminal deoxynucleotidyl transferase dUTP nick end labeling
γH2AX: H2A histone family member X
