# Supplementary figures and images for: SIRT1 regulates dermal fibroblast senescence via impaired deacetylase function and mitochondrial dysfunction during skin aging induced by chronic oral cadmium exposure
Source: Front Public Health. 2026 Feb 24;14:1779372. doi: 10.3389/fpubh.2026.1779372 (PMC12971920; doi:10.3389/fpubh.2026.1779372)

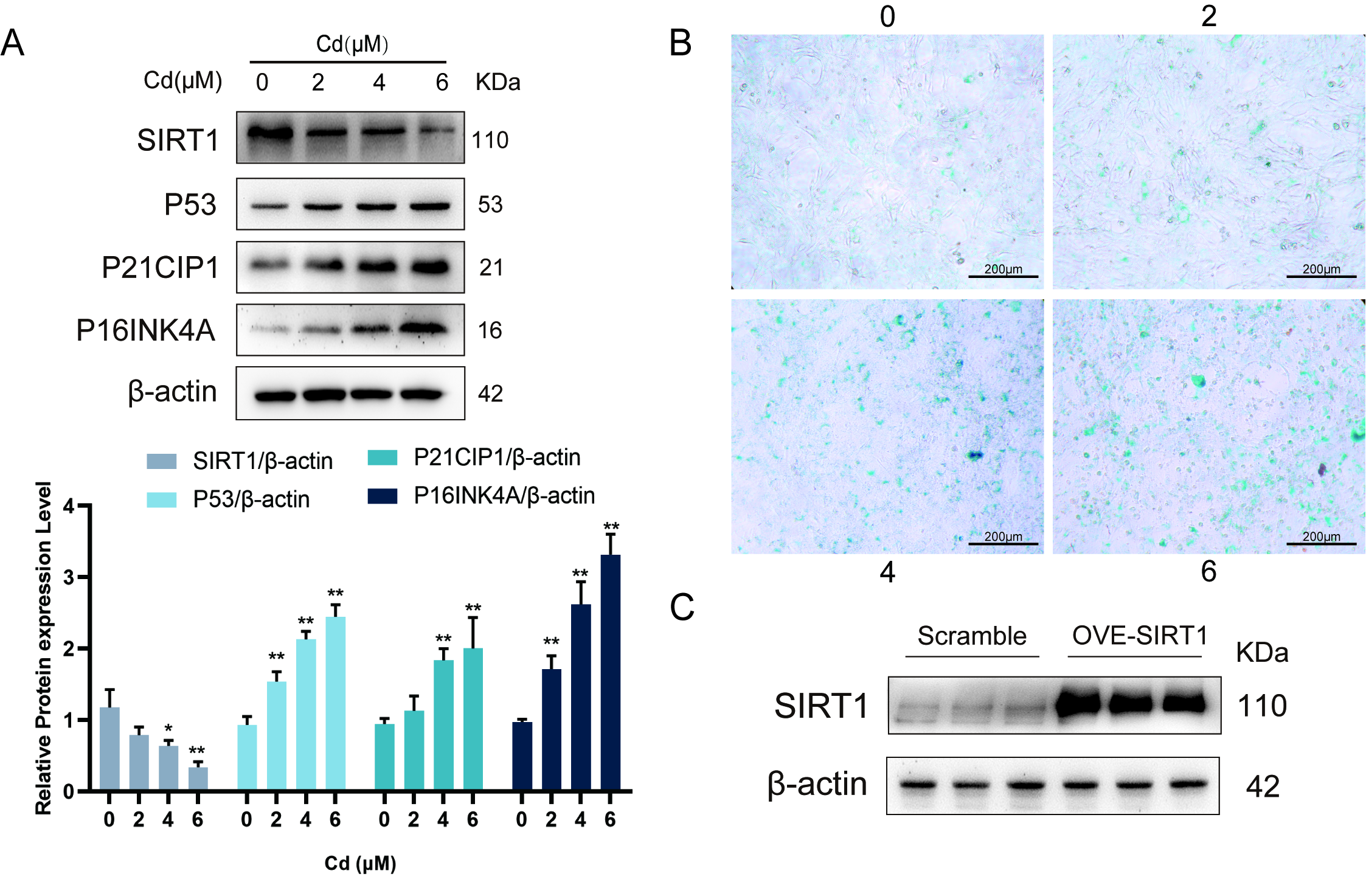

Supplement: SUPPLEMENTARY FIGURE S1 — Effects of Cd on senescence induction in C3H cells and validation of SIRT1 overexpression in vivo. (A) Western blot analysis of senescence-associated proteins (including SIRT1, P53, P21CIP1, and P16INK4A) in C3H cells exposed to increasing concentrations of Cd (0, 2, 4, and 6 μM). (B) Senescence-associated β-galactosidase (SA-beta-gal) staining demonstrating an increased proportion of senescent cells with rising Cd concentrations (0, 2, 4, and 6 μM). Scale bar = 200 μm. (C) Validation of SIRT1 overexpression in the skin tissues of Sprague-Dawley rats following AAV-r-SIRT1 administration, confirming the successful upregulation of SIRT1 protein levels in vivo. Independent experiments were performed in triplicate. Quantitative results are expressed as the mean ± SD. *P < 0.05, **P < 0.01 vs. control; #P < 0.05, ##P < 0.01 vs. Cd group. [file Supplementary_Figure_1.TIF]
